# Outcomes From Using Mortality, Antimicrobial Consumption, and Vaccine Use Data for Monitoring Endemic Diseases in Danish Swine Herds

**DOI:** 10.3389/fvets.2019.00041

**Published:** 2019-02-22

**Authors:** Ana Carolina Lopes Antunes, Vibeke Frøkjær Jensen, Nils Toft

**Affiliations:** Division for Diagnostics and Scientific Advice–Epidemiology, National Veterinary Institute/Center for Diagnostics, Technical University of Denmark, Kongens Lyngby, Denmark

**Keywords:** surveillance, monitoring, early warning, health data, swine

## Abstract

The aim of this study was to assess the potential of using multiple data sources currently available in Denmark for monitoring swine diseases. The study included farms that, based on serology, changed from “negative” to “positive” status for Porcine Reproductive and Respiratory Syndrome (PRRS), enzootic pneumonia (*Mycoplasma hyopneumonia*), and porcine pleuropneumonia (*Actinobacillus pluropneumoniae*) between January 2014 and September 2017. These corresponded to 45 swine farms working as individual operation units (i.e., their disease status is independent from other farms) and 81 farms that were part of joint operation units (i.e., 2 or more farms considered to be an epidemiological unit, having swine and personnel are transferred among them, that have the same disease status). Additionally, a total of 95 farms with a negative status for these three diseases during the study period were also included in the study. Changes in mortality data, antimicrobial consumption, and vaccine use at herd level were monitored using Shewhart control charts prior to, during, and after these farms were found positive for the three diseases. The analysis was run separately for the different age groups–weaners (up to 30 kg), sows and finishers herds–within each farm. Briefly, the highest percentage of herds generating alarms was generated up to 3 months before they changed their disease status based on mortality (30%) and 1 month after based on antimicrobial use for respiratory diseases (100%). Porcine pleuropneumonia showed to be the disease with the highest impact on these data at herd level; alarms based on the three data streams were generated in the same month that herds changed their status to porcine pleuropneumonia-positive, as well as the following months. Alarms based on vaccine use generally occurred within the same month or after changes in disease status. False alarms were found in 2% (median value) of the herds for the different age groups based on mortality and antimicrobial use for respiratory diseases in healthy farms. Monitoring changes in mortality data, antimicrobial consumption, and vaccine use showed changes (i.e., warnings) at herd level prior to confirmation from diagnostic tests.

## Introduction

Disease monitoring and surveillance involve the ongoing process of collecting and interpreting data to assess the health and disease status of a population (1). This is of paramount importance in order to reduce the impact of outbreaks and to avoid trade restrictions. The ability to detect changes in disease occurrence depends to a large extent upon the choice of data source (2).

Active surveillance implies collecting data for a specific purpose and requires significant economic resources. However, continuous monitoring of existing animal health data records is a growing field, providing a more cost-effective alternative to collecting primary data (2).

In Denmark, data about the livestock population, productivity, and health are collected on an ongoing basis (3). The current Danish databases that include swine-related data cover different aspects of animal health data, including changes in infectious disease status in terms of endemic disease in subpopulations, drug use, and mortality. These data are stored in several public and industry-owned databases.

The Specific pathogen free (SPF) system is a voluntary health program in Denmark with established rules for biosecurity, trade and monitoring of atrophic rhinitis, enzootic pneumonia, porcine pleuropneumonia, porcine reproductive and respiratory syndrome (PRRS), swine dysentery, mange, and lice within farms with an SPF certificate (4). Disease monitoring is based on clinical examination, blood samples, and nasal swabs for SPF diseases. Any farm is defined by a single location with its own unique Central Husbandry Register (CHR) number; each farm may have multiple herds each defined by species and age group, and could include animals from different owners. The visits are conducted by veterinarians from the industry Pig Research Center–SEGES and are performed on a regular basis according to the farm type. A SPF health status is assigned to the farms based on the diagnostic test results, declaring either presence of or freedom from a specific SPF disease. The designations “Red,” “Blue,” and “Green” are used to classify farms according to their biosecurity status and are used for trade within the SPF system. SPF farms represent about 40% of all Danish swine farms: 99% of Danish breeding herds have a Red-SPF status (the remaining 1% have a Blue-SPF status or are not part of the SPF system), 78% of all Danish sow herds have a Red or Blue-SPF status (the remaining 22% are not part of the SPF system), and 35% of finisher herds have Blue-SPF status (the remaining 65% are not part of the SPF system)

All Danish farmers, as well as other European farmers, are obliged to send their cadavers to rendering plants for food safety and traceability purposes (5). This regulation ensures a continuous data flow of farm mortality data that is centralized and which constitutes a strong basis for a surveillance system. The farm mortality is referred to as “mortality” throughout the manuscript.

There has been increasing concern about the use of antimicrobials in food-animal production and the emergence of antimicrobial resistance (6). The VetStat database was implemented in Denmark with the following objectives: to monitor the consumption of drugs in animal production; as a tool for veterinarians as farm advisors; to provide transparency and compliance with legislation, and to provide data for research (7). It is mandatory to register prescription data on all purchases of prescription-only drugs for production animals, either passively (by pharmacies and feed mills at the point of sale) or actively (by veterinarians, mostly related to the billing process).

In SPF herds, vaccines are used to control disease spread in case of outbreaks. Danish SPF-herds are tested on a regular basis based on serological test results for several endemic diseases (4). Danish SPF farmers are reluctant to vaccinate their animals if they are disease-free due to: (1) the risk of using vaccine-like PRRS virus (PRRS type 2 was introduced in Denmark by live modified vaccine) (8) and (2) obtaining a disease-positive status based on serological tests (the existence of antibodies can be caused by a natural infection or vaccine usage) which will results in trade restrictions. Therefore, it is reasonable to assume that the number of vaccinated herds can be used as proxy of the number of infected farms.

Further research into combining different data sources in order to improve the monitoring of swine disease is needed (9). Previous studies have demonstrated the potential use of mortality data for this purpose (10, 11). An association has also been shown between disease occurrence and an increase in antimicrobial use in Danish swine herds (12–14). However, the potential use of these data for disease monitoring and surveillance purposes at herd level remains unexplored.

The aim of this study was to explore the usefulness of multiple data sources in the monitoring of swine diseases. Changes in mortality data, antimicrobial consumption, and vaccine use at herd level were monitored in Danish breeding herds that became positive for Porcine Reproductive and Respiratory Syndrome (PRRS), enzootic pneumonia (*Mycoplasma hyopneumonia*) and porcine pleuropneumonia (*Actinobacillus pluropneumoniae*). These diseases were included in this study because they are endemic in Denmark and continue to contribute toward the economic losses associated with mortality in piglets, respiratory problems in weaners and finishers, and reproductive problems in sows. Mortality and antimicrobial and vaccine usage data were included in the study because they are potential indicators of disease outbreaks and available at farm level.

## Materials and Methods

### Mortality Data

In Denmark, swine mortality data are calculated by the Danish Veterinary and Food Administration based on the Central Husbandry Register (CHR) and the Swine Movement Database (SMD). The CHR is the national database on farm demographics, and all farms are registered. A farm is defined as a single location with its own unique CHR number. The number of swine in the three different age groups—weaners (up to 30 kg), sows and finishers (>30 kg)—is registered for each farm. The SMD includes information on all movements of swine in Denmark. Each age group within a farm is individually referred to as “a herd” throughout the manuscript. The date and number of dead finishers and/or sows transported from a farm to the rendering plant are registered in the SMD. The number of small and medium containers (with room for seven and nine weaners, respectively) is registered. It is assumed that the containers for weaners are close to full capacity at the time of transport.

Mortality data from December 2013 to September 2017 were provided by the Danish Veterinary and Food Administration. Information about movements from farms to rendering plants registered in the SMD was used to estimate the number of dead animals for each farm. The monthly mortality was calculated for each age group as a proportion: the number of dead animals in a given herd divided by the number of animals recorded in the CHR for that same herd. We have referred to this proportion as “mortality” throughout the manuscript.

### VetStat Data

All purchases of antimicrobials and vaccines made between December 2013 and September 2017 for Red-SPF farms with changes in their SPF disease status were included in the analysis. Records include detailed information such as the date, CHR number of the receiving farm, target species, age group, and disease group at the time of prescription. Internal validation showed inconsistency between age group and species in <1% of records for the included farms. These errors were corrected based on a comparison of species registered on the farm and the information on the prescription record (which included species, age group, and medicinal product) when possible, and otherwise deleted. The data were further cleaned by deleting the negative records of purchases of antimicrobials along with their corresponding positive records; negative records are mostly entered when prescribed medicines are not collected at the pharmacy, but may not contain the same degree of details as the corresponding positive record.

Antimicrobial use was measured in number of Animal Daily Doses (ADD) in order to have a measurement of comparison among the herds (15); ADD_kg_ defines the dose of treatment for 1 kg animal bodyweight.

The monthly amount of antimicrobials consumed per animal within a herd (ADD_kg_/pig^*^days _herd, month_) was calculated as described by Vigre et al. (13) for each herd (i.e., finishers, sows, and weaners). Briefly, the average daily use of antimicrobials within the herd was calculated based on the number of days between consecutive purchases, assuming that all antimicrobials purchased were used at a constant rate between purchases. A limit of 90 days was used when the time between consecutive purchases was longer than this. The data were then aggregated per month and the number of pigs registered in the CHR for each age group in each herd was used to calculate the amount of antimicrobial usage per pig-day. The consumption duration for the last purchase recorded for each herd was calculated using the average ADD_kg_/pig^*^days _herd_ from previous purchases, which differed from the approach used by Vigre et al. (13). Furthermore, the standard dose for each herd–ADD_kg_/pig^*^day _herd, month_-was then divided by an assumed average live weight of 50 kg for finishers, 200 kg for sows, and 15 kg weaners (15), which corresponds to a conversion of ADD_kg_ to standard dose ADD for an animal in the given age group. These values were calculated for the total amount of antimicrobials used (i.e., including all disease groups) and for antimicrobials purchased for the respiratory disease group alone.

Prescriptions of vaccines for either PRRS, enzootic pneumonia, or porcine pleuropneumonia for Red-SPF farms with changes in their disease status were also included in the analysis. The monthly usage of vaccines in number of doses per animal-day within a herd each month (Units/pig^*^day _herd, month_) for a specific disease was estimated as described above for antimicrobials, with the exception that there was no standardization based on the live weight. The vaccines used for a specific disease was monitored in herds that became positive for the same specific disease.

### Specific Pathogen Free (SPF) System Data

Within the SPF system, letters are sent to farmers each time a change happens to the SPF status of their farm. These changes on the SPF status happen when: (1) farms with a previously negative status receive a positive diagnostic test result or purchase animals from positive farms; (2) following partial or total depopulation-repopulation for SPF diseases; (3) when farms change their biosecurity status from “Red” to “Blue”/”Green” and vice versa. When a farm joins the SPF system, the veterinarian working for the SPF system evaluates the trading patterns and transfer of personnel with other farms. In case of regular trading and transfer of personnel among 2 or more farms, they are considered to operate as epidemiological units within the SPF system and only 10 blood samples per farm are needed for monitoring disease status. These farms are referred to throughout the manuscript as joint operation units. A change in disease status for a farm that is part of a joint operation unit will result in a change in disease status for all of the farms within that unit. In other cases, where farms do not have regular trading and transfer of personnel with other farms, these are defined as individual operation units (i.e., their SPF status is completely independent from the status of other SPF herds).

Diagnostic test results are available within 1–3 days after the blood samples collected from swine are received in the laboratory. Few herds might be re-tested within 1–2 weeks after a positive diagnostic test result in herds previously free from diseases if requested by the farmers and/or the veterinarian. In this case, the diagnostic test results from the second sampling will be used to confirm the presence of disease in the herd. Thus, letters notifying changes from a negative to a positive disease status could take from 1 day to 2.5 weeks based on laboratory diagnostic test results.

All letters sent to Red-SPF farms between January 2014 and September 2017 were provided by the SPF Sus (4). Data were only extracted from letters representing changes in SPF health status of a given farm. As previously described, the disease status may change due to a positive laboratory result for a previously seronegative herd, or due to purchases from positive farms. The letters consisted of Word document files from which metadata (including date of creation, farm CHR no.–and herd number (within CHR) when different herds have different owners–and change in disease status) were extracted.

Quarterly reports with lists of Red-SPF herds and their corresponding SPF disease status were used to identify farms free from diseases (i.e., healthy farms) between January 2014 and September 2017.

### Weighted Percentage of Alarms

Visual assessment and simple moving average were used to confirm the stationarity of the different data streams.

Changes in the data were monitored using Shewhart control charts (16), with thresholds defined using 2, 3, and 4 sd, as calculated for each individual data stream and applied separately. Data streams (time-series of mortality, antimicrobial usage and vaccine use) with at least three consecutive observations [i.e., no missing (NA) information among 3 months in a row] were included in the analysis and assumed to be independent. Herds were included in the study if at least one data stream (mortality, antimicrobial consumption, or vaccine use) fulfilled this criteria. All observations available for a given data stream (i.e., from the first record to the last) were used to calculate the different thresholds. The data were analyzed for a given month, with the month in which herds changed their disease status used as the reference month (month 0). The months before and after the reference month will be referred to as “prior” and “after” throughout the manuscript.

The weighted percentage of alarms for a given month (*WPA*_*i*_) was calculated as:

$$\begin{array}{l} WPA_{i}=\;\frac{\sum_{x.herd}\frac{x_{i}}{a_{j}}}{n.herd} \end{array}$$

Where *x*_*i*_ is the presence of alarms for month *i* using the different thresholds calculated based on Shewhart control charts, *a*_*j*_ is the sum of alarms generated for each herd *j* from the first to the last month with observations for a given data stream, and *n.herd* corresponds to the total number of herds with observations for a given month *i* and for a given data stream.

The WPA is calculated for a given month, a given data stream and a given threshold. The *n*.*herd* included in the study represents the total number of herds with observations (i.e., with average values ≥ 0 for antimicrobials and vaccines or values ≥0 for the mortality data stream), assuming these herds were active. For the vaccines and antimicrobial use, inactive herds with no observations (i.e., missing values between first and last purchase) were not included in this estimate (this was not relevant for mortality and total antimicrobial use).

Separate analyses were run for Red-SPF herds operating as individual vs. joint operation units.

### Weighted Percentage of False Alarms (WPFA)

Mortality data, as well as data on antimicrobial consumption and vaccine use for healthy Red-SPF farms was used to calculate the false alarm rate generated by Shewhart control charts. Thresholds were defined using 2, 3, and 4 sd, calculated for each individual data stream and applied separately. Data streams with consecutive monthly records from January 2014 to September 2017 (i.e., 45 data month points) were included in the analysis and assumed to be independent. The WPFA was quantified based on the WPA described above with *x*_*i*_ corresponding to the absence of alarms for month *i* using the different thresholds calculated based on Shewhart control charts.

All methods were implemented in R (version 3.3.3) (17).

## Results

A total of 53 letters sent to individual operation unit farms and 40 letters sent to farms within joint operation units were included in the analysis.

Table 1 describes the number of Red-SPF farms and herds with changes in their disease status, as well the corresponding number of records in the different databases within the study period (January 2014–September 2017). The number of Red-SPF farms that became positive for Enzootic pneumonia was higher both for individual and joint operation units when compared to herds that became positive for PRRS and porcine pleuropneumonia during the study period.

**Table 1:** Description of the number of Red-SPF farms with changes in their disease status between January 2014 and September 2017, and the corresponding number of herds with registrations within this time period for mortality, antimicrobial consumption, and vaccine use for different age groups.

**Disease**	**Data stream**	**Individual operation unit**			**Joint operation unit**		
		**Weaners**	**Sows**	**Finishers**	**Weaners**	**Sows**	**Finishers**
PRRS	Total number of farms		12			25	
	Mortality	9	5	12	20	9	12
	Total antimicrobial consumption	8	5	12	17	8	14
	Antimicrobial consumption for respiratory diseases	4	5	3	3	5	4
	Vaccines for PRRS	0	1	0	4	4	0
Enzootic pneumonia	Total number of farms		14			41	
	Mortality	11	8	13	22	15	41
	Total antimicrobial consumption	11	7	12	26	15	40
	Antimicrobial consumption for respiratory diseases	8	5	6	11	12	11
	Vaccines for Enzootic pneumonia	2	5	2	17	16	11
Porcine pleuropneumonia	Total number of farms		6			5	
	Mortality	4	4	6	2	2	3
	Total antimicrobial consumption	4	3	6	3	2	4
	Antimicrobial consumption for respiratory diseases	4	3	5	5	1	2
	Vaccines for Porcine pleuropneumonia	1	1	0	2	1	2

*A farm is defined as a single location with its own unique CHR number and a herd is defined as individual age group within a farm*.

Monitoring the total antimicrobial consumption did not generate alarms for herds in either type of operation unit.

**Figure 1:**
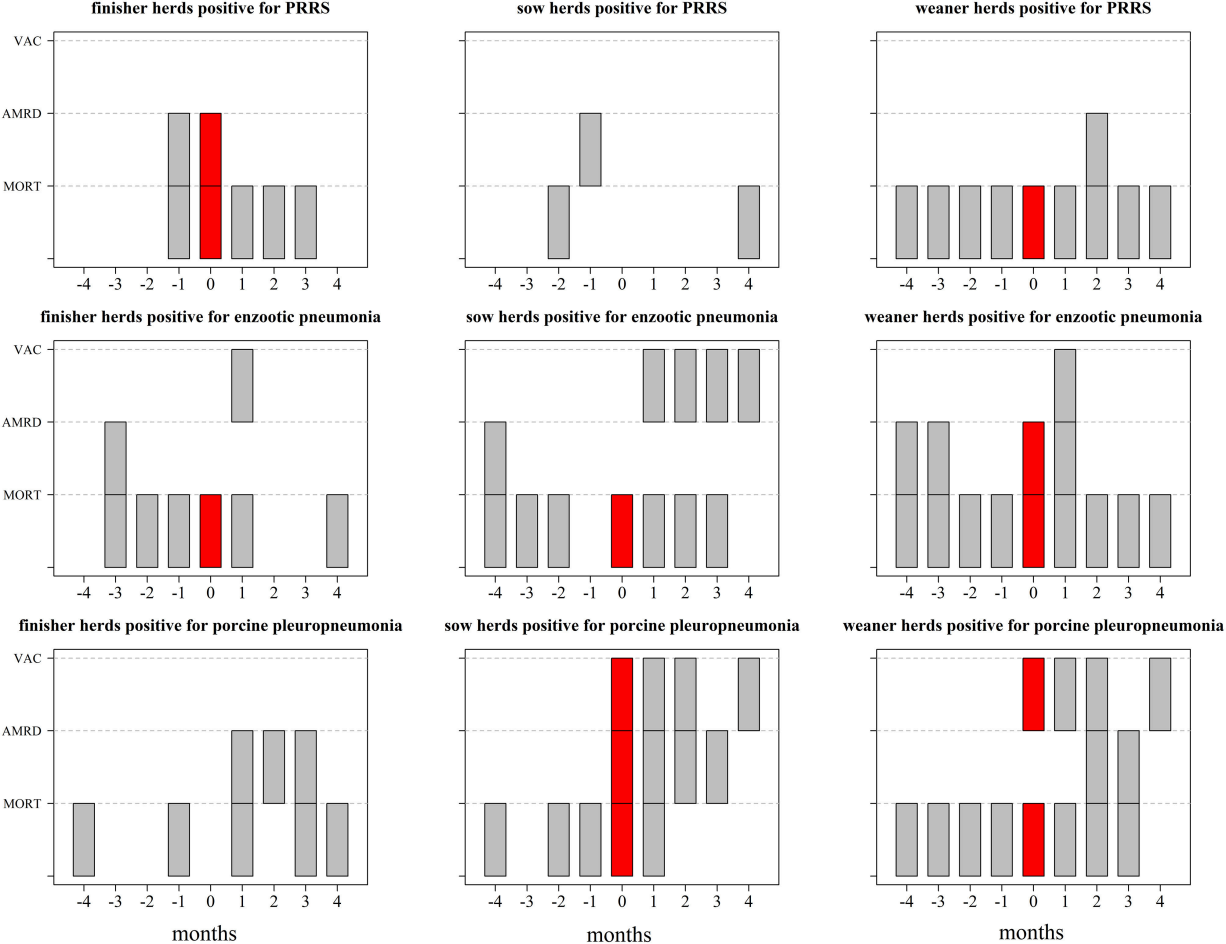
Description of which data streams generated alarms in at least one herd based on Shewhart control charts using 3 sd prior to, during, and after changes in disease status (month = 0) in farms that function as individual operation units between January 2014 and September 2017. The data streams included mortality at farm level (MORT), antimicrobial consumption for respiratory diseases (AMRD), and vaccine use (VAC). The results are based on 12 farms that became positive for Porcine Reproductive and Respiratory Syndrome (PRRS), 14 farms that became positive for enzootic pneumonia and 6 farms that became positive for porcine pleuropneumonia; the corresponding number of herds can be found in Table 1. Each gray or red “block” represent the existence of at least 1 herd with alarms in a given month (x axis) generated based on mortality (MORT), antimicrobial consumption for respiratory diseases (AMRD), and/or vaccine use (VAC) (y variables); the inexistence of blocks (white space) refers to the inexistence of alarms or no data available [in the case of vaccine use (VAC)]—please refer to Table 1.

**Figure 2:**
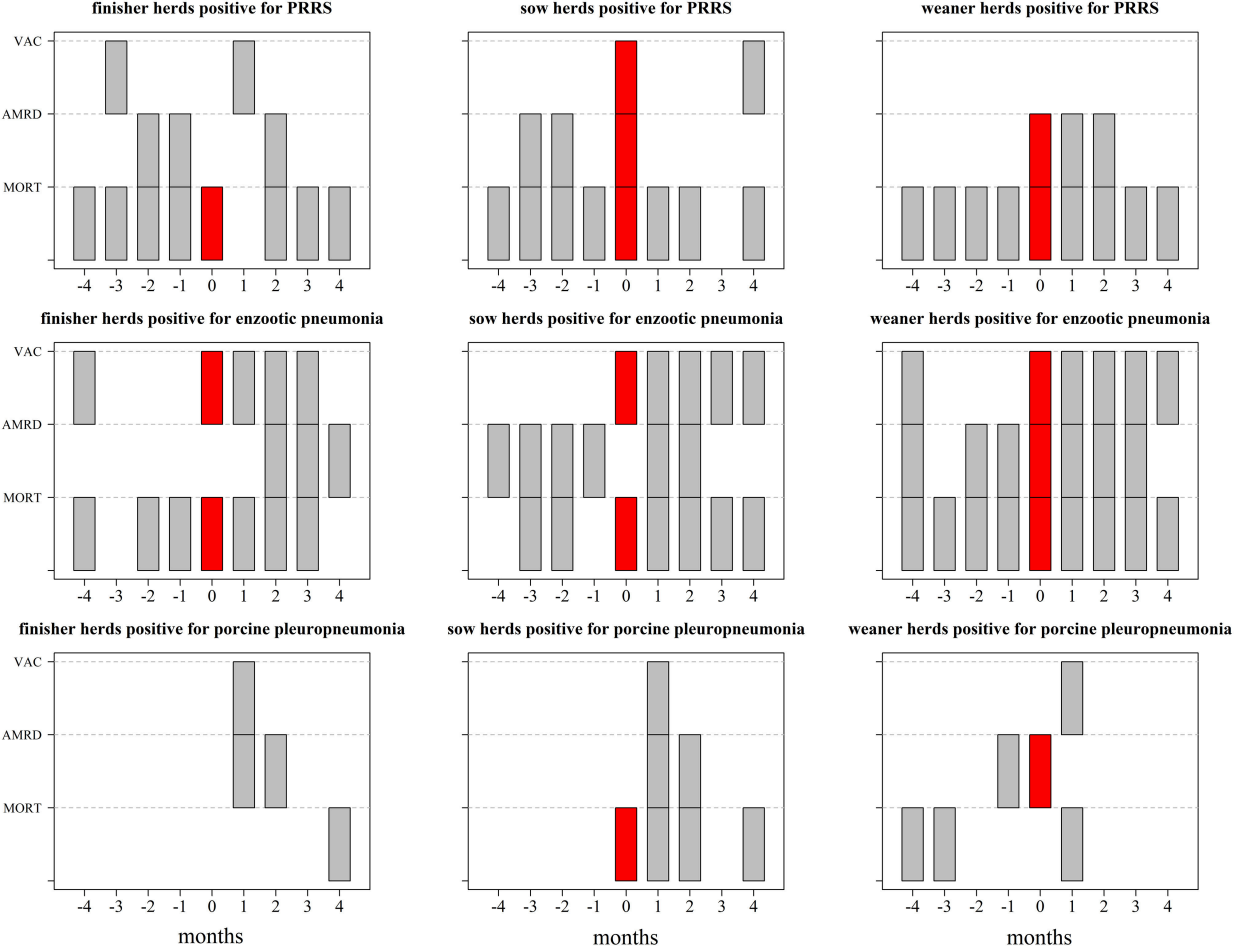
Description of which data streams generated alarms in at least one herd based on Shewhart control charts using 3sd prior to, during, and after changes in disease status (month = 0) in farms that function as joint operation units between January 2014 and September 2017. The data streams included mortality at farm level (MORT), antimicrobial consumption for respiratory diseases (AMRD), and vaccine use (VAC). The results are based on 25 farms that became positive for Porcine Reproductive and Respiratory Syndrome (PRRS), 41 farms that became positive for enzootic pneumonia and 5 farms that became positive for porcine pleuropneumonia; the corresponding number of herds can be found in Table 1. Each gray or red “block” represent the existence of alarms in a given month (x axis) generated based on mortality (MORT), antimicrobial consumption for respiratory diseases (AMRD), and/or vaccine use (VAC) (y variables); the inexistence of blocks (white space) refers to the inexistence of alarms or no data available [in the case of vaccine use (VAC)]—please refer to Table 1.

Figures 1, 2 show which databases generated alarms, during, and after changes in their disease status for both individual and joint operation units based on 3sd, representing the middle threshold used in the analysis. For both types of operation units, alarms based on vaccine use generally occurred in the same month (i.e., month 0) as changes in their status (Figures 1, 2).

For individual operation units, an increase in alarms based on mortality was seen for sow herds up to 2 months prior, and an increase in alarms based on antimicrobial use for respiratory diseases was seen 1 month prior to their disease status becoming PRRS-positive (Figure 1). For enzootic pneumonia, increases in the number of alarms based on mortality and antimicrobial use for respiratory diseases in weaner, sow, and finisher herds were found up to 4 months prior. Alarms generated based on mortality data, antimicrobial consumption, and vaccine use were seen in the months during which sow and weaner herds became positive for porcine pleuropneumonia (i.e. month 0).

For joint operation units, alarms based on mortality and antimicrobial consumption for respiratory diseases were found for finisher and sow herds 3 months prior in herds that became PRRS-positive (Figure 2). The three data streams generated alarms after herds changed their disease status to positive for enzootic pneumonia and porcine pleuropneumonia (Figure 2).

**Figure 3:**
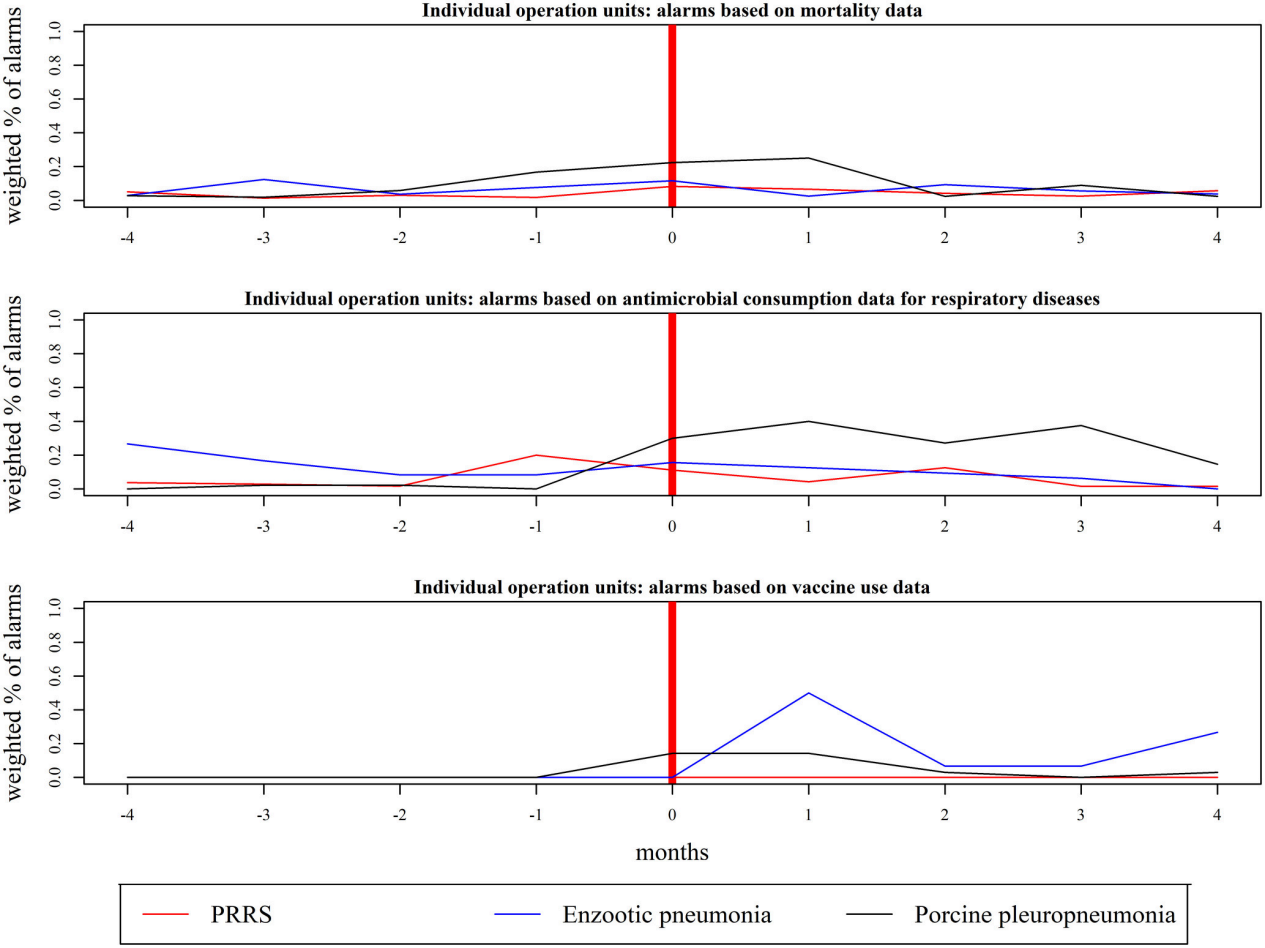
Highest weighted percentage of alarms (WPA) achieved prior to, during, and after changes in disease status (month = 0) in farms that were part of individual operation units. The alarms were found on mortality, antimicrobial consumption for respiratory diseases and vaccine use based on Shewhart control charts (using 2, 3, and 4 sd). Each line represents the highest values achieved for a given month by the 3 thresholds for a given data stream. The results are based on 12 farms that became positive for Porcine Reproductive and Respiratory Syndrome (PRRS), 14 farms that became positive for enzootic pneumonia, and 6 farms that became positive for porcine pleuropneumonia; the corresponding number of herds can be found in Table 1.

**Figure 4:**
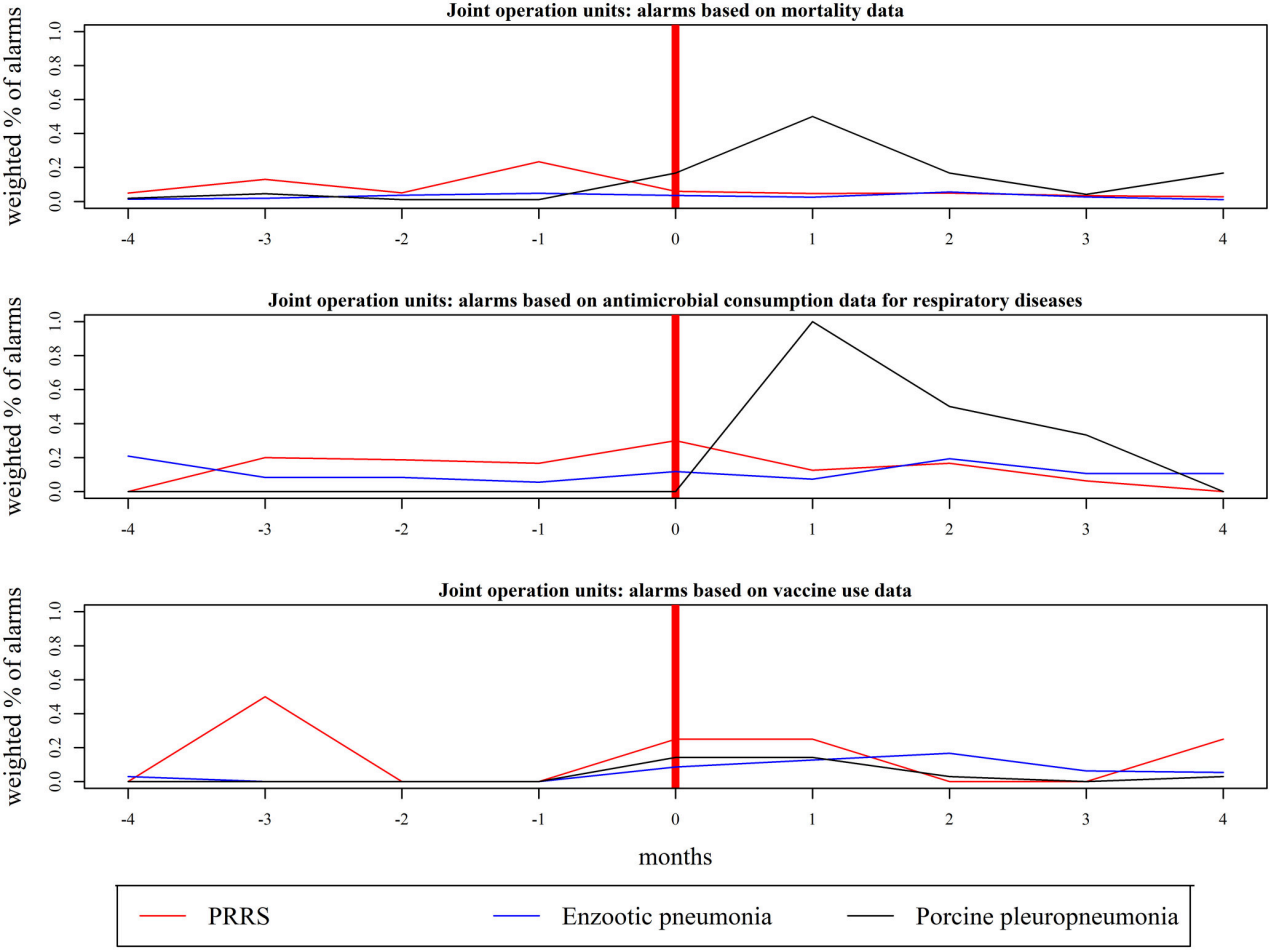
Highest weighted percentage of alarms (WPA) achieved prior to, during, and after changes in disease status (month = 0) in farms that were part of joint operation units. The alarms were found on mortality, antimicrobial consumption for respiratory diseases and vaccine use based on Shewhart control charts (using 2, 3, and 4 sd). Each line represents the highest values achieved by the 3 thresholds for a given data stream. The results are based on farms that became positive for Porcine Reproductive and Respiratory Syndrome (PRRS), 41 farms that became positive for enzootic pneumonia, and 5 farms that became positive for porcine pleuropneumonia; the corresponding number of herds can be found in Table 1.

The highest weighted percentage of alarms (WPA) achieved for a given month before, during, and after changes in herd disease status was calculated based on Shewhart control charts using 2, 3, and 4sd, as shown in Figures 3, 4. The highest WPA based on mortality for PRRS and enzootic pneumonia were found in month 0 (WPA = 0.08) and 3 months prior (WPA = 0.12), respectively; an increase from WPA = 0.02 3 months prior to WPA = 0.25 1 month after was seen for herds that became positive for porcine pleuropneumonia (Figure 3). The highest WPA based on the consumption of antibiotics for respiratory diseases for PRRS, enzootic pneumonia, and porcine pleuropneumonia was achieved between 1 month prior and 1 month after (WPA = 0.20 for PRRS, WPA = 0.4 for porcine pleuropneumonia and WPA = 0.16 for enzootic pneumonia), while for vaccine use, these values were achieved in month 0 for porcine pleuropneumonia (WPA = 0.14) and month 1 after the change for enzootic pneumonia (WPA = 0.5) (Figure 3). For joint operation units, the highest WPA based on mortality was observed for PRRS 1 month prior (WPA = 0.23; Figure 4). For vaccine use, the highest WPA for PRRS was achieved prior to changing disease status (3 months prior, WPA = 0.5) (Figure 4). For enzootic pneumonia and porcine pleuropneumonia, the highest values of WPA were achieved for months ≥ 0 based on the three data streams; a WPA = 1 was achieved 1 month after the change to a porcine pleuropneumonia-positive status based on antimicrobial consumption for respiratory diseases (Figure 4).

**Figure 5:**
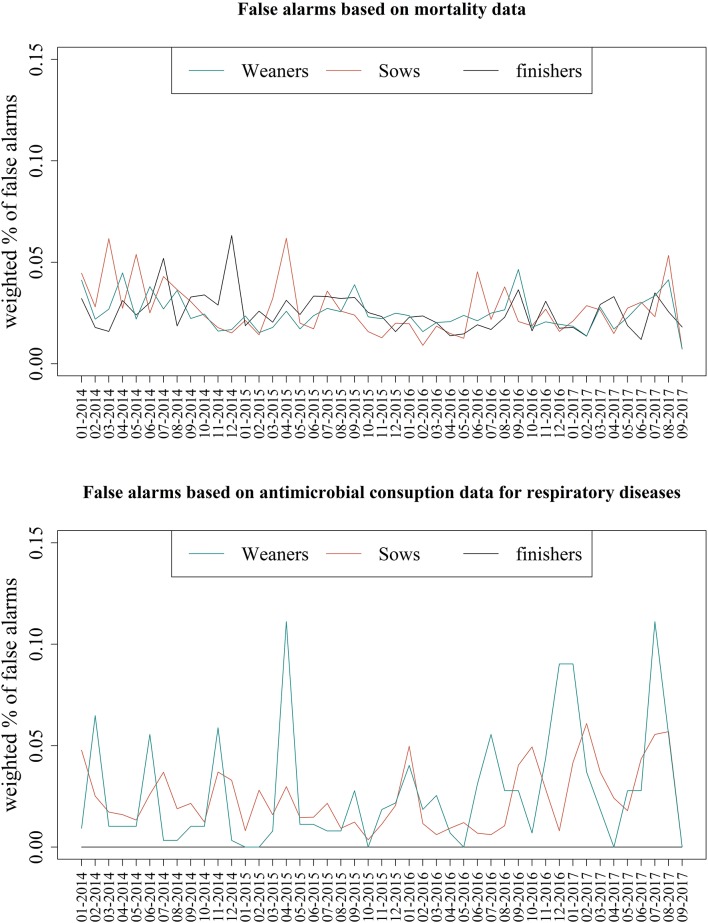
Highest weighted percentage of false alarms (WPFA) found in data from Red SPF-herds negative for Porcine Reproductive and Respiratory Syndrome, enzootic pneumonia, and porcine pleuropneumonia between January 2014 and September 2017. The alarms were found on mortality, antimicrobial consumption for respiratory diseases, and vaccine use based on Shewhart control charts (using 2, 3, and 4 sd) and they are based on 95 farms; the corresponding number of herds can be found in the Results section.

A total of 95 healthy Red-SPF farms was included in the study. The number of weaner herds with records for mortality, antimicrobial consumption, and antimicrobial consumptions for respiratory diseases was 56, 60, and 18, respectively. For sow herds, a total of 50 herds for mortality, 55 for antimicrobial consumption, and 27 for antimicrobial consumption for respiratory diseases were included in the study. Finisher herds represented 69 herds with records for mortality, 61 herds for antimicrobial consumption, and 2 for antimicrobial consumption for respiratory diseases. No registrations of use of vaccines for PRRS, enzootic pneumonia, and porcine pleuropneumonia were found in these farms, except 1 finisher herd with registers of purchase of vaccine against porcine pleuropneumonia in 2 consecutive months (not included in the study). A WPFA>0 was found for all herds based on mortality and antimicrobial consumption data for respiratory diseases in the entire study period (Q1 ≤ 0.02, median = 0.02, Q3 ≥ 0.03), except for consumption data for respiratory diseases in finishers (WPFA = 0) (Figure 5). A WPFA = 0 was found when monitoring changes on the total antimicrobial consumption of these herds. Regarding the use of vaccines for PRRS, enzootic pneumonia and porcine pleuropneumonia, only a single finisher herd had records of vaccine usage for porcine pleuropneumonia during 6 months (Figure 5).

## Discussion

This study explored the potential of using different data sources currently available in Denmark to monitor swine diseases. Changes in mortality data, antimicrobial consumption, and vaccine use at herd level were monitored in Red-SPF farms prior to, during, and after these farms became positive for Porcine Reproductive and Respiratory Syndrome (PRRS), enzootic pneumonia (*Mycoplasma hyopneumonia*), and porcine pleuropneumonia (*Actinobacillus pluropneumoniae*). Based on mortality and antimicrobial use for respiratory diseases, the highest values for WPA based on mortality (up to 30%) were generated up to 3 months before herds changed their disease status. Porcine pleuropneumonia showed to be the disease with the highest impact (i.e., increases in mortality and antimicrobial consumptions) at herd level, with 100% of herds generating alarms 1 month after changing to a positive status. In general, most alarms based on the three data streams were generated in the same month in which herds changed their status to porcine pleuropneumonia-positive, as well as the following months. Alarms based on vaccine use generally occurred within the same month or after changes in disease status. False alarms were found in 2% (median value) of the herds for the three age groups based on mortality and antimicrobial use for respiratory diseases in healthy farms.

We decided to analyze the data by age group due to the physiological differences between these groups. For example, a higher mortality would be expected in weaners compared to other age groups due to factors relating to nutrition, thermal stress, and disease (18). Additionally, the amount and pattern of antimicrobials prescribed in Danish swine farms also differed between age groups (19).

As these diseases are part of the porcine respiratory disease complex (PRDC), i.e., pneumonia of multiple etiologies causing clinical disease (20), we chose to monitor changes in antimicrobial consumption for respiratory diseases. The total consumption of antimicrobials at herd level (for a given age group) was also included in the study due to possible misclassification of the disease group registered in VetStat, or the potential decision to use antimicrobials prescribed for other diseases to treat respiratory symptoms. The vaccine use was included in the study to explore the potential of using these data as proxy for disease occurrence. This is important for the Danish food and veterinary administration that own the mortality and prescription data but have no access to the letters sent to SPF farms (data owned by the industry) and to the laboratory data from farms in general (owned by the farmers). Vaccines are used to control disease spread in case of outbreaks and the number of vaccinated herds can be used as proxy of the number of infected farms; however, it is also possible that some farms vaccinate their animals prior to e.g., export as a requirement, or have a vaccine program that lasts several months (seen mainly for joint operation units that became positive for enzootic pneumonia—Table 1). It should be noted, that vaccination strategies may differ in herds that are not part of the sero-surveillance (non-SPF herds). Within the SPF system, some farmers will decide to start eradication programs based on total/partial depopulation—repopulations, without vaccines, due to the trade restriction imposed to seropositive herds. We also found herds with a single purchase of vaccines, but we decided not to include these data in the study as we have no information on when they were administrated to the animals.

We looked at the 4 months prior to and 4 months following changes in the disease status of a herd as we assumed that other diseases might be more likely to explain changes in the data outside of this period. The reason why time-series with at least 3 data points were included in the study was based on the assumption that vaccine and (the majority) of antimicrobials for treatment of outbreaks will not be used on regular basis (as it would for prophylactic purposes), and therefore, its usage will be limited in time (few months).

The low values for WPA commonly observed in the results indicate that alarms were raised in a minority of the herds (<50%), which may be explained by the varying impact these diseases have within a herd. Some farms may be subclinically infected with PRRS, while others may experience severe reproductive and/or respiratory disease with high mortality (20). This also depends on the strain of disease, dosage of treatment, and the immune status of the herd. Although enzootic pneumonia is usually a chronic and mild disease, it can cause an increase in the severity of PRRS-induced pneumonia (20). Outbreaks of porcine pleuropneumonia may occur in a peracute form resulting in sudden death, or as a subacute/chronic form with a cough, increased antibiotic use, lower daily gain and poor feed conversion, and exercise intolerance (18, 20). Furthermore, the availability of control measures such as vaccination or health-management programs and the high biosecurity status of Danish breeding farms (i.e., with a Red-SPF status) contribute to a lower incidence rate when compared to exotic or (re-) emerging diseases. It is therefore unlikely that “extreme” changes in incidence and prevalence would be observed for diseases already present in Denmark.

A larger number of data streams generated alarms and increased WPA values in the months after changes (seroconversion) were observed in joint operation units. This might be because it is only necessary to have a single farm with a positive diagnostic test result or purchases from positive farms in order to change the disease status of all farms within the joint operation unit. Subsequently, the transfer of personnel and animals among these farms makes it likely that disease will spread over time and have an impact in these farms at a later stage (i.e., months after the change in their disease status). The same rationale can be used to explain the higher number of alarms for joint operation units occurring after changes in their disease status. In contrast, individual operation units represent single farms that have received laboratory confirmation of the presence of disease, or that have purchased animals from positive farms, which would have an impact at herd level prior to or in the same month as the positive diagnostic test results, thus generating higher WPA in these periods.

The SPF health status is defined at farm level except if herds belonging to the same farm have different owners (which is very uncommon). When using the data from the CHR and movement database (both used to calculate mortality data) and Vetstat data registered per CHR no. (i.e., farm level), bias can occur. If the same farm have several herds (e.g., different owners or different housings) within the same age group, it is more likely to have both healthy and sick animals, resulting in lower mortality and antimicrobial consumption at farm level and, consequently a lower number of alarms.

It is assumed that the containers for weaners are close to full capacity at the time of transport because the costs of rendering are paid by the farmers. Furthermore, the fact that the Danish Veterinary and Food Administration uses these data for welfare control purposes might motivate farmers to fill the containers to full capacity as more containers (i.e., more dead weaners) are perceived as an indication of welfare issues.

Running separate analyses for the individual operation units (each with positive laboratory results) and joint operation units (where the majority of the farms have a healthy status but will eventually develop the disease from an infected farm in the same unit) indicated that the latter showed a lower weighted percentage of alarms (WPA) prior to changes in their disease status. The CHR data were used as a proxy for the number of animals of each age group (herd) present in a farm for any given month. This information is updated in the database at least once/twice per year by farmers or the SEGES Pig Research Center (21). It is therefore possible that a dynamic herd of varying size might be misrepresented in the CHR, and as a consequence, changes in the denominator we used to calculate mortality, antimicrobial consumption, and vaccine use could be biased.

As described in a previous article (11), registering dead weaners (<30 kg) based on the number of containers (with specific dimensions) transported from a farm to the rendering plant may lead to bias in the data and consequently contribute to false alarms (or a lack of alarms).

When considering antimicrobial consumption and vaccine use, it is important to note that VetStat registers the date that drugs are purchased and not the date of consumption. It was assumed that all antimicrobials and vaccines purchased were used at a constant rate between two consecutive purchases or within a specific period of time. This might represent a limitation as it does not take into account variation among veterinarians, the frequency of diseases, dosages, duration of treatments, or fluctuation in prices. As per the legislation, it is legal to prescribe antimicrobials for diseases expected to arise in the period before the next veterinary consultation—usually 1 month. Antimicrobial prescription may be due to an outbreak prior to the visit, as antimicrobial on farm's stock may have been used. However, the farmers usually only have medicines on stock that are expected to be used before the next visit, because it is administratively laborious to have medicines on stock for longer periods, because it has to be signed by the veterinarian at every visit (due to legislation).

The presence of false alarms in healthy Red-SPF farms evidenced the presence of variation (noise) in the data, which might be caused by bias in registers, herd management and the presence of other diseases not tested as part of the SPF system. However, this is also a result of the trade-off between sensitivity and specificity; if no false alarms are accepted, the true alarms will be harder to detect and vice-versa. Moreover, the Shewhart control chart is used to monitor “peaks” in data streams (16) and it was applied directly to the modified stationary time-series. This method does not allow to monitor (very) gradual shifts in the data and only accounts for peaking shifts in time series. As previously discussed, the diseases included in the study can cause severe or mild cases. In the latest case, very gradual increases in mortality and on antimicrobial consumption might occur and no alarms based on the Shewhart control chart will be generated.

In this study, retrospective data available for a given time period was used. The data in the different databases are retrieved at different paces: (1) the laboratory serology test results are updated every day within a couple of minutes when the laboratory diagnostic test is available; (2) the mortality data are calculated once a month by the Danish food and Veterinary Administration (based on data updated on daily basis in the CHR and swine movement database) and (3) the data on purchases in VetStat are available within between 3 and 45 days (22). This range of reporting time and possible delays will be a bottleneck when a monitoring system is set up in Denmark based on these data.

More detailed studies are needed to determine whether the changes in these data streams also reflect other types of Danish swine herds, such as another type of SPF farm or non-SPF farms, where the prevalence of endemic diseases is higher compared to Red-SPF farms (8, 23). Additionally, other monitoring methods more suitable for monitoring gradual changes in data (with missing observations) for multivariate monitoring need to be evaluated.

## Conclusions

Monitoring changes in mortality data and antimicrobial consumption for respiratory diseases showed changes (i.e., alarms) at herd level prior to diagnostic test confirmation. These results also show a potential value in using these data streams for monitoring diseases and in the development of monitoring and surveillance strategies at a national level by the Danish government.

## Ethics Statement

The study was conducted using registered data and did not involve experiments on animals. From an ethical perspective, all of the data collected and used as part of this study was outside the scope of Directive 2010/63.

## Availability of Data and Materials

The datasets used and analyzed in the current study are owned by the Danish Veterinary and Food Administration and the SPF Sus–SEGES.

## Author Contributions

AL contributed to the study design, data management, and analysis and wrote the manuscript. VJ and NT assisted in designing the study and reviewed the manuscript.

### Conflict of Interest Statement

The authors declare that the research was conducted in the absence of any commercial or financial relationships that could be construed as a potential conflict of interest.
